# Diffusion-free valve for preprogrammed immunoassay with capillary microfluidics

**DOI:** 10.1038/s41378-023-00568-2

**Published:** 2023-07-17

**Authors:** Pooya Azizian, Jasmina Casals-Terré, Jordi Ricart, Joan M. Cabot

**Affiliations:** 1 Energy and Engineering Department, Leitat Technological Center, Terrassa, Barcelona Spain; 2 Mechanical Engineering Department, Technical University of Catalonia, Terrassa, Barcelona Spain

**Keywords:** Electrical and electronic engineering, Chemistry

## Abstract

By manipulating the geometry and surface chemistry of microfluidic channels, capillary-driven microfluidics can move and stop fluids spontaneously without external instrumentation. Furthermore, complex microfluidic circuits can be preprogrammed by synchronizing the capillary pressures and encoding the surface tensions of microfluidic chips. A key component of these systems is the capillary valve. However, the main concern for these valves is the presence of unwanted diffusion during the valve loading and activation steps that can cause cross-contamination. In this study, we design and validate a novel diffusion-free capillary valve: the π-valve. This valve consists of a 3D structure and a void area. The void acts as a spacer between two fluids to avoid direct contact. When the valve is triggered, the air trapped within the void is displaced by pneumatic suction induced from the capillary flow downstream without introducing a gas bubble into the circuit. The proposed design eliminates diffusive mixing before valve activation. Numerical simulation is used to study the function and optimize the dimensions of the π-valve, and 3D printing is used to fabricate either the mould or the microfluidic chip. A comparison with a conventional valve (based on a constriction-expansion valve) demonstrates that the π-valve eliminates possible backflow into the valve and reduces the mixing and diffusion during the loading and trigger steps. As a proof-of-concept, this valve is successfully implemented in a capillary-driven circuit for the determination of benzodiazepine, achieving the successive release of 3 solutions in a 3D-printed microfluidic chip without external instrumentation. The results show a 40% increase in the fluorescence intensity using the π-valve relative to the conventional value. Overall, the π-valve prevents cross-contamination, minimizes sample use, and facilitates a sophisticated preprogrammed release of fluids, offering a promising tool for conducting automated immunoassays applicable at point-of-care testing.

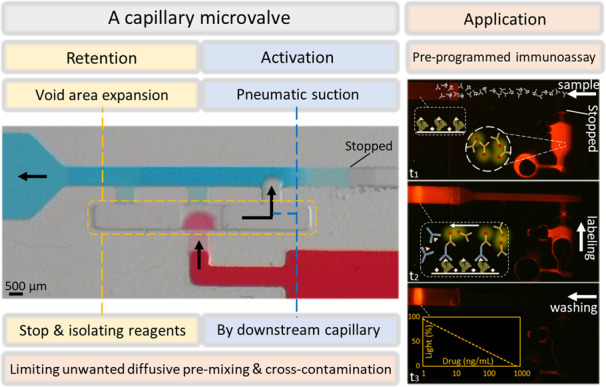

## Introduction

Over the last decade, diagnostic devices have become miniaturized, and cost-effective testing at the point-of-care (POC) has been conducted. Microfluidics has successfully contributed to the field of diagnostics, providing reliable devices, such as pregnancy tests, urine dipsticks (e.g., drug detection), and blood glucose monitoring^1,2^. The manipulation of small volumes in microfluidic devices has allowed the automation of rapid and cost-effective chemical and biological analyses, reducing the number of reagents and samples needed^3^. Due to these great advantages, microfluidics is a key enabling technology for POC testing. However, the need for robust peripheral equipment is a key limiting factor in reaching an ideal microfluidic-based POC device. Electrical and mechanical components, such as pumps and valves, are bulky and difficult to implement^4^. In addition, miniaturized methods, such as electrowetting, can be used^5–10^. However, electrowetting microvalves have limited applications due to the uncontrolled effects of charges on molecules, the requirement of embedding a supply source, and most importantly, the manufacturing difficulties for the deposition of electrode layers on the microfluidic substrate.

Recently, capillary-driven microfluidics has become a powerful alternative to move and stop fluids spontaneously without external forces. The capillary effect is driven by surface tension and adhesion between the liquid molecules and the confining walls. To reduce peripheral instrumentation, a wide variety of capillary fluidic elements have been developed over the years^11,12^. A key element to control microfluidics is the valve^13^. Capillary valves can spontaneously stop and reactivate fluids without external parts. This phenomenon can occur with an abrupt expansion of the microchannel. This expansion causes a rapid decrease in the capillary force, triggering the stop of the fluid from the front end, which is called the downstream end^12,14–19^. In some cases, the meniscus curvature can change from concave to convex momentarily, stopping the capillary flow to the trigger. Another strategy involves implementing a constriction from the back end of the fluid, which is called the upstream end. In this situation, there is a negative retention pressure that can induce capillary action and stop the flow^20,21^. The subsequent delivery of the fluid is typically activated by a secondary fluid^14,15^. Capillary valves are named differently in the literature based on their functions to stop and actuate the fluid flow. For example, capillary burst valves stop the flow upstream until the flow is triggered^22^, and capillary trigger valves stop the flow downstream until another liquid breaks the meniscus and triggers it^12,14,15^.

Microfluidics can be preprogrammed by synchronizing the capillary pressure and flow resistance values^23,24^. Juncker et al. presented capillary circuits based on the sequential delivery of liquids using retention burst valves^20^. Zimmermann et al. designed a multicompartment capillary circuit with delay valves that trigger flow when this is rejoined to the main channel^15^. In preprogrammed circuits, there are two steps for actuation of the valves: (i) breaking the meniscus at the trigger junction by capillary flow in the main microchannel and (ii) overcoming the retention pressure upstream^25–27^. Safavieh and Juncker encoded the successive flows of multiple reagents to perform an immunoassay^25^. Recently, Yafia et al. demonstrated a domino concept that serially connects multiple reservoirs to control their release sequentially^28^. This effect significantly enhances the reliability of the reagent release timing. These methods work properly for the programming and control of actuation. However, after breaking the meniscus at the trigger junction until compelling upstream retention and activation, diffusive mixing of molecules from both fluids is unavoidable. Diffusion is a real problem when using low volumes and long delivery times. Moreover, unexpected changes in the capillary pressure can cause backflow from the main fluid to the triggered reagent. Alternatively, it is possible to use a trigger channel as a mediator between the main and supply channels. In this case, when the trigger channel is empty, the valve is closed due to sudden expansion. However, when a liquid is introduced into this mediator channel, the valve is open^29^. A capillary backflow from downstream of the main channel into the narrower trigger channel can be used to activate such a valve. However, the problem of this work is that the liquid in the supply channel prefers to flow through the narrow trigger channel rather than the target main channel. Menges et al. introduced an off-valve that closes the trigger flow immediately after the actuation event to prevent unwanted flow into the trigger channel^29^. In their design, a secondary flow with a high capillary pressure pushes the air into the off-valve section, which cuts the flow connection, similar to transistor switching^30^. Although this design requires sophisticated fabrication and some reagent waste in the mediator channels, it provides the opportunity to avoid unwanted diffusion before the activation time. POC applications, such as quantitative biosensing based on competitive immunoassays, seek new capillary valves to avoid even small amounts of waste and diffusive mixing before valve activation.

In this paper, a novel diffusion-free valve is developed for preprogrammable capillary-driven microfluidics. The proposed design introduces a void area between fluids where air acts as a spacer. When the valve is triggered, the air trapped within the void is displaced by a pneumatic suction induced from the capillary flow of the main channel. The proposed design is demonstrated for the quantitative detection of benzodiazepines. The diffusion-free valve is validated in different situations and multiple architectures.

## Materials and methods

### Numerical method

Capillary-driven flow through a microfluidic device is an in-compressible multiphase flow with an immiscible liquid–air interface. We numerically modelled this system using Navier‒Stokes equations, including surface tension force, by its adaptive spatial discretization to track liquid–air interfaces^31,32^. Herein, capillarity drove the liquids. Capillarity is represented as a force applied on a meniscus (${\rm{\sigma }}{\rm{\kappa }}{{\rm{\delta }}}_{{\rm{s}}}{\bf{n}}$), where σ, κ, ${\delta }_{s}$, and n are the surface tension, curvature, Dirac delta, and normal vector to the interface, respectively^33^. In addition, a fluid resistance opposed the flow that could be modelled as a viscous term ($\nabla .(2{\rm{\mu }}{\bf{D}})$), where ${\rm{\mu }}$ and ${\bf{D}}$ are the fluid viscosity and the deformation tensor, respectively. Gerris open-source computational fluid dynamics (CFD) code was utilized for the numerical simulation^34^. SI 1 describes the governing equations and the applied numerical method details.

### Fabrication of microfluidic chips

The microfluidic devices were designed with SolidWorks 2021 (Dassault Systèmes SE, France) and printed using the stereolithography (SLA) 3D printer Form3 (Formlabs, USA). Clear V4 Resin was used in all cases. Two strategies were adopted to fabricate capillary-driven microfluidic chips: i) soft lithography using 3D printed moulds and ii) 3D printed (3DP) microfluidics sealed with pressure-sensitive adhesive (PSA). For the soft lithography process, a polydimethylsiloxane (PDMS) mixture (10:1 mixture of PDMS and its crosslinking agent, SYLGARD 184, DOWSIL) was cast on the 3D printed multilevel moulds. Subsequently, the polymer mixture was degassed and cured in an oven at 60 °C overnight. After peeling, the mixture surface was activated by oxygen plasma and subsequently rinsed with a 20% polyethylene glycol (PEG; model MW 35000, Sigma‒Aldrich) solution to enhance its surface hydrophilicity^35,36^. Finally, the hydrophilic PDMS chip was plasma bonded on a glass substrate covered by a thin layer of spin-coated PDMS (hydrophobic, nontreated). Having a hydrophobic base attached to the hydrophilic microfluidic devices permitted the capillary elements of the π-valve to function correctly. For the second and relatively fast fabrication strategy, the microfluidic chip was 3D printed and activated by atmospheric plasma (Plasmatreat Steinhagen FG3001 + RD1004, Germany). A 1.6 mm-thick optically clear silicone adhesive was used to seal the microchannels (PSA, ARclear 93495, Adhesives Research, Ireland). Similar to the PDMS chip, the 3D printed part was hydrophilic (C.A.$\sim$30°), whereas the sealing layer of PSA was hydrophobic (C.A.$\sim$100°). To minimize the effect of overpolymerization from the SLA printer, a comprehensive calibration between computer-assisted designs (CADs) and 3D-printed features was carried out. Resolution and standard deviation were analyzed for different geometry characteristics (open channels, pillars, and holes). Metrology analysis was correlated with the drawing and subsequently corrected in the CAD design. By applying this correcting factor, we achieved the desired dimensions of the 3D-printed microchips. Measurements of the water contact angle were performed regularly to assure constant hydrophilicity. The results showed a stable contact angle of 30° ± 5° for more than a week for the 3DP chips.

### Capillary-driven microfluidic experiments

For all the experiments, deionized water was mixed with 20% w/w food dye, and 10 $\mu$L of the dyed deionized water was used for the volume of each reservoir. For inspection, a smartphone camera, digital microscope (AM4115T-FUW-EDGE, Dino-Lite, The Netherlands), and inverted microscope (Euromex, Oxion Inverso 2053) were used to record the videos and take pictures. ImageJ (National Institutes of Health, http://rsb.info.nih.gov/ij/) was used to analyze the region of interest (ROI) and monitor the mean intensity value of the ROI versus time. Images were processed to split into color channels in which the black background was set at an intensity value of 0.

### Capillary pressure and flow resistance

The flow was driven by the capillary pressure due to the surface tension between the liquid and the confining walls. All the microchannels had driving and/or retention capillary pressure along or against the capillary flow. The capillary pressure could be calculated by employing the Young–Laplace equation^37^. In a closed rectangular microchannel, the capillary pressure could be stated as follows:^20,23,24^

1 $${\rm{P}}=-{\rm{\gamma }}\left[\frac{{\rm{cos }}{{\rm{\theta }}}_{{\rm{t}}}+{\rm{cos }}{{\rm{\theta }}}_{{\rm{b}}}}{{\rm{h}}}+\frac{{\rm{cos }}{{\rm{\theta }}}_{{\rm{l}}}+{\rm{cos }}{{\rm{\theta }}}_{{\rm{r}}}}{{\rm{w}}}\right]$$

where $\gamma$ and $P$ are the surface tension of the liquid and the pressure, respectively; ${\theta }_{t}$, ${\theta }_{b}$, ${\theta }_{l}$, and ${\theta }_{r}$ are the top, base, left, and right water contact angles from the respective channel walls, respectively; $h$ is the channel height; and $w$ is the channel width. According to Hagen–Poiseuille’s law, the pressure decrease that occurs due to viscous forces on the walls could be described using the flow rate (Q) and the fluidic resistance (R). For a rectangular cross-section channel, R could be calculated using the length (L) of the microchannel (Eq. 2)^26^.

2 $${\rm{R}}=\frac{\Delta {\rm{P}}}{{\rm{Q}}}=\frac{12{\rm{\mu L}}}{{{\rm{h}}}^{3}{\rm{w}}}{\left[1-0.630\frac{{\rm{h}}}{{\rm{w}}}\right]}^{-1}$$

### Competitive immunoassay of benzodiazepine

In all the immunoassays, the solutions were prepared in phosphate-buffered saline (PBS). A nitrocellulose membrane strip (Unisart® CN95) was used within the detection areas to ease protein immobilization. Benzodiazepine and anti-benzodiazepine stock solutions were spiked in 5% bovine serum albumin (BSA). BSA solution was used as the blocking buffer, and PBS was used as the washing solution. The secondary detection antibodies were conjugated with quantum dots (F(ab')2-Goat anti-Mouse IgG (H + L) Secondary Antibody, Qdot™ 605; Thermo Fisher Scientific). A Dino-Lite digital microscope was used for fluorescence detection under 405 nm excitation. In addition, we used a colored glass filter (FGL610S, 610 nm longpass, Thorlabs, Inc.) to sort wavelengths other than the quantum dot (QD) emissions. The exposure time was set at 1/8 s, and the power and gain were the same in each fluorescence detection. Detection was carried out inside a black box, and 3DP black resin (SLA, FormLabs) was used to fabricate the microfluidic chip to reduce light noise.

## Results

### π-valve function

**Fig. 1:**
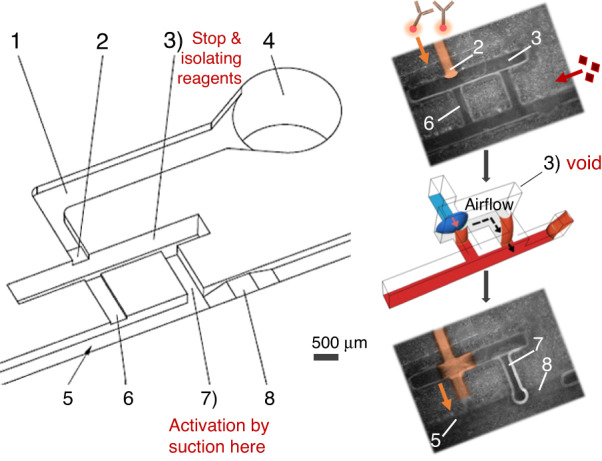
Sketch of the diffusion-free valve (π-valve). $\pi$-valve encompasses: (1) inlet channel, (2) stop junction, (3) void (air chamber), (4) reagent inlet, (5) main channel, (6) shallow branch, (7) deep branch, and (8) activation resistance component. The valve avoids diffusive mixing of the reagents by using the isolating air void before their activation. The detailed step-by-step process is described in Table 2

In microfluidics, bubbles can be a problem and must not be generated, especially when working in low-pressure devices, such as capillary-driven microfluidic devices. Bubbles can be trapped at the corners or the expansion sites of a microfluidic channel (e.g., detection sites and serpentine areas), leading to microfluidic device malfunction, poor antigen–antibody interaction, and even fluidic circuit disconnection^38^. However, trapping the air between two liquids is the most efficient method for eliminating diffusion since it acts as a spacer to avoid fluid contact. With the support of simulation tools, a novel $\pi$-shaped capillary valve is developed to deliver liquids in a preprogrammed manner without problems derived from diffusion or form introducing a gas bubble into the circuit. The structure and capillary elements of this π-valve are shown in Fig. 1.

The $\pi$-valve comprises an inlet channel where reagent is introduced, a void to create the air gap, and two channels (named deep and shallow branches) connected to the main channel. The deep branch controls the pressure of the trapped air in the void, and the shallow channel is designed to move the reagent from the inlet to the main channel. The π-valve is triggered by activation resistance component, which is a retention valve located in the main channel. This retention is sufficiently high to activate the release of the reagent contained in the $\pi$-valve. To find the right dimensions of each capillary element, a range of possible dimensions is evaluated. Then, the capillary pressure and flow resistance are calculated from Eq. 1 and Eq. 2 and tested in silico with the support of the simulation tools. The optimum depth and width/depth relationships are listed in Table 1.

**Table 1:** Dimensions of π-valve components

	Depth (*μ*m)	Width/Depth ratio
Main channel	400	1.75
Deep branch	400	1.25
Shallow branch	100	5
Activation resistance	100	7
Void	900	0.83
Stop junction	200	2.5

To further clarify the $\pi$-valve design, the valve is described as a combination of singular microfluidic elements and their functions. (i) A stop valve formed by the inlet channel (1), stop junction (2), and void (3). The stop valve works based on a downstream expansion to stop the flow. (ii) A retention valve made of the main channel (5) and the activation resistance component (8). The retention valve is based on an upstream constriction to hold the liquid. (iii) A connection between the aforementioned stop valve (i) and the retention valve (ii). This connection is formed by the shallow branch (6), deep branch (7), and void (3); it activates the stop valve when the retention valve holds the flow in the main channel. The connection feature is designed to replace the air gap from the void into the deep branch. This air replacement is crucial to avoid introducing an air bubble into the shallow branch and subsequently into the downstream circuit, leading to possible flow malfunction. The functional steps and the activation process are categorized and explained below based on the resulting illustrations.

The loading and activation process of the π-valve consists of 5 sequential steps: reagent loading, air trapping, equilibration, activation, and release. We list the characteristics of the function and activation in Table 2 to better understand them. This table includes a short description of the process, illustrations from the simulation (CFD phase tracer), experimental pictures, and pressure contours derived from CFD. The first step of the π-valve loading and activation process, reagent loading, consists of filling the valve with the reagent liquid. The reagent flows spontaneously by capillary action until it reaches a sudden expansion, the stop junction (2). This phenomenon follows the same principle as the stop valves. The second step, air trapping, starts when the second liquid is introduced from the inlet of the main channel. This liquid flows along the main channel, passing through the activation resistance component (8). Subsequently, this liquid fills the deeper branch (7) and the shallow branch (8) before continuing through the main channel (5). The second liquid does not fill the void chamber (3) due to the sudden expansion of the void. Unlike the conventional trigger valves, the second liquid does not come into contact with the reagent until the activation step. In the third step, equilibration, the pneumatic pressure created in the void chamber slightly pushes the liquid of the deep branch back to the main channel. As the flow resistance and capillary pressure of the shallow branch are higher than those of the deep branch, the air is displaced to the right, where the capillary force is relatively low. This movement is small and can be difficult to detect. The fourth step, activation, occurs when the second liquid in the main channel is emptied up to the activation resistance component (8) just before the $\pi$-valve. Since the activation resistance is higher than the equivalent resistance of the π-valve, the capillary flow from the main channel provides suction power to empty the liquid from the deep branch (7), which has a relatively low fluidic resistance. Consequently, the pneumatic pressure moves the air trapped in void (3) to deep branch (7), activating the entrance of the reagent liquid from inlet channel (1) to void (3). The meniscus of the reagent liquid in the stop junction (2) changes from concave to convex due to the capillary pressure. In the fifth and final step, release, the reagent liquid connected with the end of the second liquid at the shallow branch (6). Finally, the reagent flows along the main channel (5). No bubbles are created during this step. The pressure contours from Table 2 depict the pressure changes in the void areas, stop junctions, and fluidic resistance components. The pneumatic void pressure increases by trapping the air due to the capillarity of the liquids within the branches. The pressure reaches equilibrium at void that is ~200 Pa greater than the atmospheric (atm) pressure. CFD results reveal that the dimension of the activation resistance should be chosen in such a manner that a two-phase meniscus provides a capillary pressure of ~650 Pa (e.g., a 100 × 700 μm section), while the deep branch has the same depth as the main channel. It is necessary to decrease the trapped air pressure by ~300 Pa under atm through the deep branch to move the trapped air and open the valve. While 1 cm downstream in the main channel provides 1 kPa capillary suction. Video 1 (SI) shows the activation and release process of the $\pi$-valve by tracing the phases using computational fluid dynamics. The transient dynamics of the two-phase interfaces are depicted in the video. Liquids fill the microchannels and move downstream by capillarity at the curved meniscus, which comes from adhesion to the walls. The contact angles with the walls clearly show the hydrophobicity of the lower wall and the hydrophilicity of the rest. The trapped air and its dynamics can be seen in the void by following the movements of the interfaces. Video 2 and Video 3 (SI) show the $\pi$-valve experimental activation and release using PDMS and 3D-printed microfluidic chips, respectively. Moreover, SI 2 and Video 4 depict a modified π-valve, changed in favor of low-resolution 3D printers.

**Table 2:** Steps of the loading and activation processes of the π-valve

Process Step	Simulation	Experimental Image	Pressure Contour
Loading – The first liquid (blue) was loaded from the inlet channel until the stop junction before entering to the void area. The second liquid (red) passed through the activation resistance component in the main channel.			
Air Trap – As soon as the second liquid (red) filled the deep and shallow branches, the air trapped in the void isolated the blue and red liquids.			
Equilibration – The capillary action from the shallow branch increased the pressure within the void. To equilibrate this pneumatic pressure, the liquid (red) in the deep branch was pushed slightly back to the main channel.			
Activation – The first liquid (blue) was active when the second liquid (red) emptied up to the activation resistance in the main channel. Since the activation resistance was strong, the capillary pressure in the downstream could not empty the liquid from the main channel, providing the required suction power to the deep branch. Subsequently, the air trapped in the void was displaced by the pneumatic suction, which introduced the blue liquid into the void.			
Release – As a result, the air displacement connected the blue liquid with the red liquid. The blue liquid was released spontaneously to the main channel through the shallow branch.			

The simulation pictures were taken from Supplementary Information Video 1, showing the phase contour between the air–liquid phases. The experimental pictures are screenshots from Supplementary Information Video 2, showing the autonomous release of blue liquid triggered by red liquid. Pressure contour pictures were collected from the computational fluid dynamics calculations that were determined by following the governing equation mentioned in SI 1

### Electric analogy

**Fig. 2:**
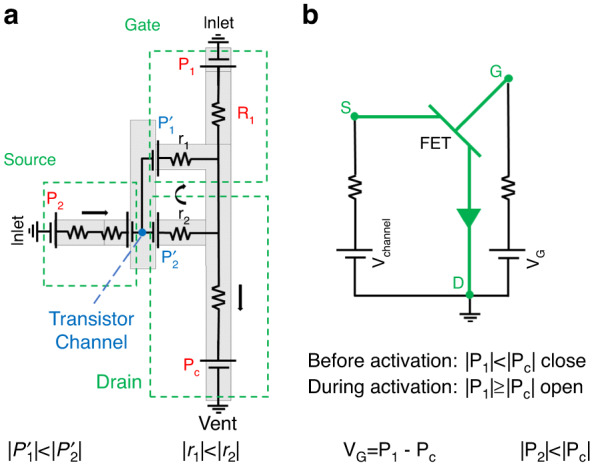
Conceptualization of the π-valve and its operations in the form of its nearest electronic equivalent (FET). **a** Electronic analogy of the π-valve. The meniscus is a negative potential source. The liquid flows toward the potential with the lowest magnitude. ${{\rm{P}}}_{1}$ and ${{\rm{P}}}_{2}$ are the capillary retention pressures upstream of the main channel and branch, respectively; P_c_ is the capillary pressure downstream; ${{\rm{R}}}_{1}$, ${{\rm{r}}}_{1}$, and ${{\rm{r}}}_{2}$ are the activation resistance, deep branch, and shallow branch, respectively; G, S and D are the typical terminals of a transistor, the gate, the source and the drain, respectively; ${{\rm{V}}}_{{\rm{G}}}$ is the gate voltage; and ${{\rm{V}}}_{{\rm{c}}}$ is the channel voltage. **b** Transistor circuit analogy. The void operates like the channel of a FET. Conditional equations from the figure must be considered for correct operation. When the gate potential is sufficiently large to stop the current downstream in the main channel, its field effect on the void pressure opens the transistor channel. Consequently, the valve is activated

For robust filling and activation, each microfluidic element of the π-valve is necessary. Often, capillary microfluidic elements are associated with individual electric components and arranged to make an electric analogy view. Figure 2 is the conceptualization of the valve to the nearest electronic equivalent, which is a field-effect transistor (FET). Capillary pressure (Eq. 1) can be defined as a potential source in the electrical analogy, whereas the fluidic resistance (Eq. 2) and the flow rate are equivalent to the electric resistance and the electric current, respectively. The meniscus is considered a negative potential source. Generally, the liquid flows spontaneously to the negative potential with the largest magnitude. During the loading step, the magnitude of the capillary pressure downstream, |${P}_{c}$ | , is larger than the magnitude of the retention pressures upstream, |${P}_{1}$ | . Consequently, the liquid flows downstream through the main channel, which fills the two branches of the π-valve. As soon as the liquid reaches the second branch, the capillary circuit between the valve and the main channel is connected by the void. This component works as the channel of the transistor. At this stage, the electric resistance of the transistor channel is infinite due to void expansion; this component functions similar to a semiconductor located between the source and drain electrodes. This resistance totally drops, and the channel of the transistor is opened by applying a relatively large potential to the gate of the transistor. When the liquid is emptied up to the activation resistance in the main channel (${R}_{1}$), the magnitude of the retention pressure (${P}_{1}$) significantly increases as the channel depth decreases. This process is similar to that of changing the voltage of the transistor gate, which leads to a field effect in the transistor channel and opens it to a current between the source and drain. That is, when the retention pressure is powerful enough that capillary action cannot suck the liquid in the very shallow flow resistor, its field effect decreases the pressure in the void and triggers the fluid flow at the multilevel stop and opens the valve.

### Diffusion analysis

**Fig. 3:**
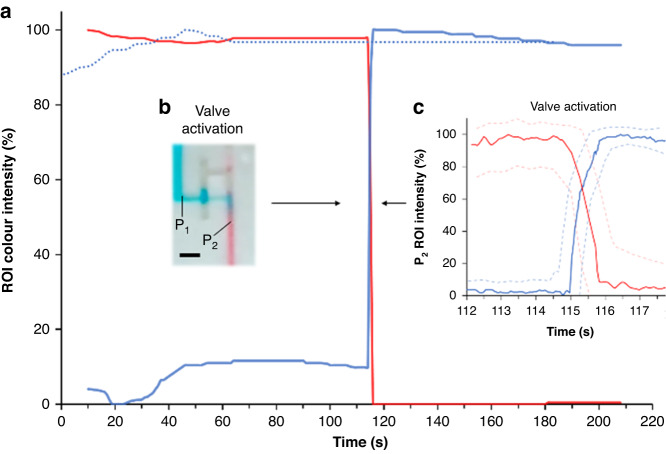
The valve diffusion analysis using the colour intensity evolution. **a** Color intensity during π-valve loading and activation. Solid red and blue lines are the color intensities of the red and blue liquids at position ${P}_{2}$, respectively. The dotted blue line is the color intensity of the blue liquid at position ${P}_{1}$. **b** Picture of the valve during activation. The scale bar is 1 mm. **c** Color intensity during activation at position 2 (${P}_{2}$). The solid lines are the average intensity at 20 Hz, and the dashed lines are their respective standard deviations. Background noise is subtracted by selecting a second ROI close to the detection site. Intensities are taken from Video 3 (SI), which shows the activation and release of the 3D printed $\pi$-valve

To study the diffusion of molecules from one reagent to the other, blue and red intensities are monitored at two different points (${{\rm{P}}}_{1}$, ${{\rm{P}}}_{2}$) throughout the process. ${{\rm{P}}}_{1}$ is 1 mm upstream from the stop junction of the void, and ${{\rm{P}}}_{2}$ is 1 mm downstream from the connection between the shallow branch and the main channel. Figure 3 shows the intensity of the two dyes and a picture of the positions of ${{\rm{P}}}_{1}$ and ${{\rm{P}}}_{2}$. Clearly, ${{\rm{P}}}_{1}$ shows no diffusion during the 2 min before valve activation and after release. In addition, no backflow into the branch side is observed. By using the void, diffusion is avoided. At activation, the blue intensity at ${{\rm{P}}}_{2}$ increases to 100% in 0.8 s. However, the speed of the release is highly correlated with the suction pressure of the capillary pump or an absorbable pad positioned at the end of the main channel. This valve reduces the volume mixed down to 450 ± 60 nL (*N* = 5). The volume is calculated by multiplying the area where the blue and red liquids are mixed by the channel depth. After activation, mixing is negligible because the upstream flow from the main channel is stopped beforehand.

**Fig. 4:**
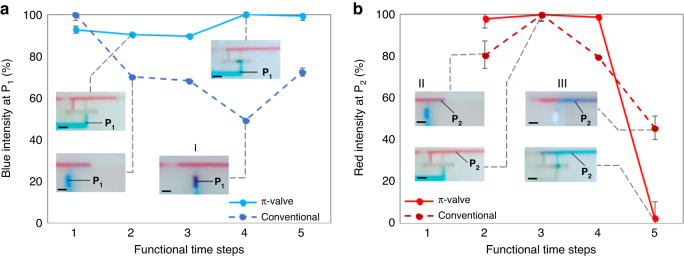
Comparison of the diffusion of liquids between π-valve and conventional valve (based on constriction-expansion). Functional time steps are defined as (1) loading; (2) air trapping/interface breaking; (3) air equilibration/pressure equilibration; (4) activation/trigger; and (5) release. **a** Percentage of blue intensity at P_1_. **b** Percentage of red intensity at P_2_. P_1_ is located 1 mm upstream from the stop junction of the void, and P_2_ is located 1 mm downstream from the connection between the shallow branch and the main channel. Backflow into the reservoir is observed for the conventional valve (I). Furthermore, the reagent diffuses into the main channel before valve activation (II), and mixing occurs when the valve is released (III). No mixing is observed for the $\pi$-valve before release. The scale bars are 1 mm

Conventional capillary valves are based on meniscus rupture by a secondary fluid. To activate multiple valves, such as during the preprogramming release of reagents, diffusion or backflow into the reservoirs is unavoidable due to their various upstream retentions. Therefore, to prove the benefit of the π-valve, a comparison with conventional valves for the 5 process steps is carried out and is shown in Fig. 4. The trigger channel of the conventional valve has the same dimension as the shallow branch of the $\pi$-valve and a retention of ~400 Pa (300$\times$500 µm channel) upstream to have a similar timing as the $\pi$-valve. In both devices, 3D printed chips with 10 µL reservoirs are used, and the intensity is monitored at the same ${{\rm{P}}}_{1}$ and ${{\rm{P}}}_{2}$ positions. In step 2, the conventional valve experiences a 20% decrease in intensity in ${{\rm{P}}}_{2}$. Subsequently, due to the backflow, the red reagent is slightly pushed into the reservoir and diffused with the blue reagent (steps 2, 3, and 4 at ${{\rm{P}}}_{1}$). Diffusion is visible in step 4, where the intensity is reduced by 50% at position ${{\rm{P}}}_{1}$. However, the π-valve does not experience any change in intensity. During the release of the valve (step 5), the red intensity in position ${{\rm{P}}}_{2}$ does not reach 0% as quickly as the $\pi$-valve. The previous backflow and diffusion into the reservoir modified the composition of the reagent within the reservoir. For a reservoir with such a small volume (10 µL in both cases), diffusion can be significant. Considering the small volumes used, the need for retention burst valves to synchronize multiple retentions is a difficult task and relies on users’ ability. For example, excess solution can result in an inefficient burst valve causing undesired release of minute amounts of solution from reservoirs. One of the advantages of this new valve is that its function relies less on the users and instruments than the other valve. In contrast, the π-valve shows more promising control and less failure than the other valve because of the air barrier between reagents.

SI 3 presents how in a conventional valve, overloading or deficient loading of reagents leads to backflow into the reservoir or convection into the main current, respectively. While π-valve not only evades these loading deficiencies, but also offers better sequencing by limiting dead volumes and precise activation time (Video 5). Neither maximum overloading of the entrance nor placing it against gravity can fail π-valve retention. In addition, the larger void expansion than the junction expansion at the main microchannel keeps the π-valve functional for using surfactant concentrations 4 times greater than that used in the conventional valve. Moreover, SI 3 shows an analysis of the valve impacts on the flow pattern through the sensing area with and without nitrocellulose. The detection site medium and geometry affect fluid-diffusive phenomena, such as Taylor dispersion. These characteristics control the flow rate and profile, aiming to proceed toward a lateral flow pattern as much as possible. However, tracing color intensities in the detection site shows that the valve impacts on having a downstream lateral flow pattern are as essential as using the area characteristics and reagent properties to suppress dispersion and are even more desirable for exact sequencing.

### Preprogrammed capillary-driven microfluidics with ${\boldsymbol{\pi }}$-valves

**Fig. 5:**
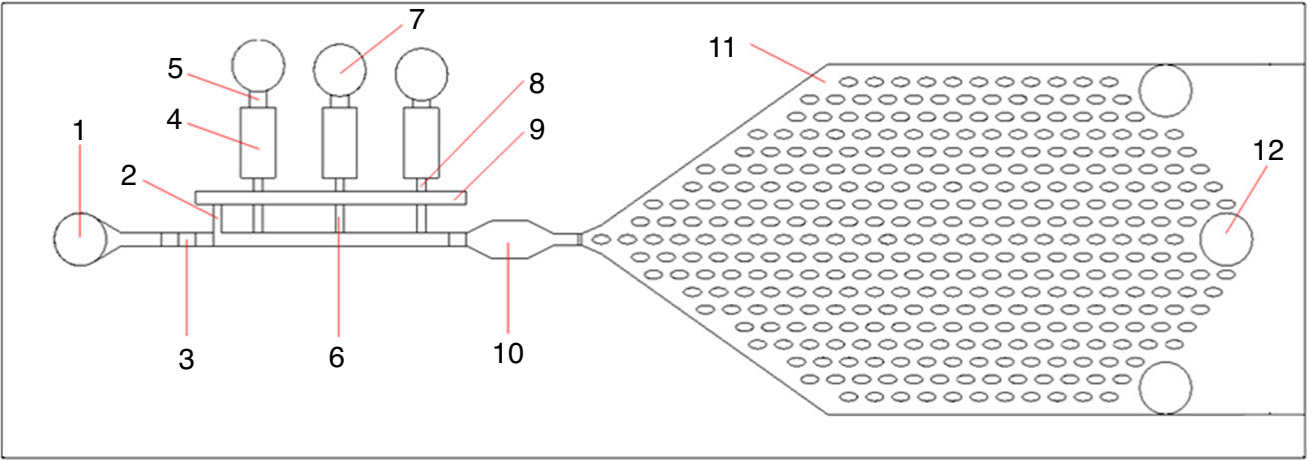
Sketch of the preprogrammed π-valve array within a capillary circuit. The components are the (1) inlet of the main channel, (2) deep branch, (3) activation resistance, (4) reagent reservoir, (5) retention burst, (6) shallow branch, (7) reagent inlet, (8) stop junction, (9) void area, (10) detection site or reaction chamber, (11) capillary pump, and (12) air vent

Certain applications, such as immunoassays, require a sequential introduction of reagents and rinsing steps^21^. Therefore, the activation and release of multiple fluids must be preprogrammed in a single and autonomous device. For these types of applications, two strategies for sequencing π-valves are designed and tested. For the first strategy, $\pi$-valves are connected in parallel to the main channel using different voids. SI 4 presents this strategy for two parallel valves. However, this strategy increases the required activation suction pressure. This strategy can lead to unwanted corner flow, which weakens the valve function. Therefore, for cases that require multiple π-valves, it is recommended to share a single void for all reagents. This second and more reliable strategy prevents the system from requiring high burst pressures. Figure 5 shows a sketch of 3 π-valves sequenced in a single autonomous device. In this design, the pneumatic suction from a deeper branch acts as the trigger of all the reagents stopped in the junctions. The array of $\pi$-valves is integrated within an automated capillary circuit that encompasses (i) different inlets to introduce the reagents; (ii) vent to displace the air; (iii) reservoirs to store the reagents; (iv) flow resistance components to control the flows; (v) $\pi$-valves to stop and reactivate the fluid flows; (vi) a sensing/reaction chamber; and (vii) and a capillary pump. The capillary pump is designed considering previous studies^39^. By assigning different retention burst pressures upstream (element 5 in Fig. 5; magnitude of the retention pressures from left to right: 374 Pa, 290 Pa, and 249 Pa), it is possible to preprogram the release of the 3 fluids. Listed in Table 3 is the release of 3 colored dyes with the process of each step. The retention burst pressure of the blue chamber is less than that of the green chamber and the red chamber. The retention burst pressures reversely depend on the channel dimensions, where the shallow channel has a relatively high retention level. Therefore, precise automation of the sequential releases is achieved by synchronizing the retention pressures and capillary pump suction. Video 6 (SI) shows the experiments using color dyes through the multiplied $\pi$-valve fabricated by 3DP and sealed by PSA.

**Table 3:** Process steps of the preprogrammed release of liquid with a single void

Process Step	Experimental Result
Loading – The liquids were loaded through reservoir inlets and stopped at the multilevel stop junctions just before the void. The liquid stopped due to the fluidic resistance levels of the abrupt expansions. The surface tension allowed long storage times (days) without activation or evaporation when maintained in water-saturated ambient.	
Air Trap & Equilibration – The trigger liquid (red) was introduced through the main channel, filling the deep branch and the 3 shallow branches. The air was trapped in the void to isolate the 3 liquids from that in the main channel. The red liquid continued through the detection site and the capillary pump.	
1st Activation – The branch with the smallest retention burst (blue) was activated when the red liquid in the main channel emptied to the activation resistance component. The downstream capillary pressure provided sufficient suction power to activate the blue liquid. The air in the void was displaced by the pneumatic suction.	
2nd Activation – The same principle occurred for the activation of the chamber with the intermediate retention burst (green). When the 1st chamber was emptied, the next π-valve was activated using the next shallow branch available (closest to the reservoir).	
3rd Activation – The last release was the one with the highest retention burst (red). The red liquid was activated and flowed into the main channel through the last shallow branch. Backflow or diffusion was not apparent during the sequential release of the liquids.	
Detection – The capillary pump provided enough suction power to empty the 3 different fluids. However, it was not strong enough to overcome the activation resistance. Therefore, the liquid remaining in the main channel was not emptied. This phenomenon allowed the measurement to be carried out in wet and homogenous conditions.	

The scale bar is 2 mm

### Immunoassay within the capillary-driven circuits

As a demonstration, two capillary-driven circuits (with and without the π-valve) are developed for the determination of benzodiazepine. The determination of this drug is performed by a competitive immunoassay. First, 0.5 µL of drug-BSA in PBS, with a concentration of 1.4 mg/mL, is drop-casted in the middle of the nitrocellulose membrane and integrated within the microfluidics detection site. The immobilized drug-BSA is used to capture the free antibodies coming from the sample. If the anti-drug antibodies are fluorescent, the concentration of the drug in a sample is inversely proportional to the fluorescence intensity.

**Fig. 6:**
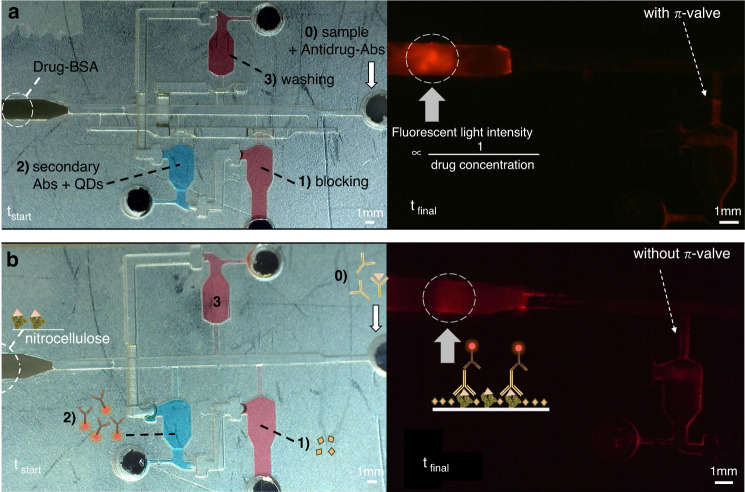
The microfluidic sequence for benzodiazepine detection. Competitive immunoassay for benzodiazepine detection using capillary-driven microfluidic devices with a $\pi$-valve array (**a**) and without a $\pi$-valve array (**b**). The determination consists of an activation step (step 0) and 3 subsequent flow events. The trigger step (0) starts with the introduction of 30 µL of sample with the anti-drug in the main channel (5 µg/mL anti-benzodiazepine in 5% BSA without benzodiazepine). In step (1), the solution of reservoir 1, which is filled with 10 µL of 5% BSA for blocking, was eluted and passed through the detection zone. Subsequently, in step (2), reservoir 2, which is filled with 10 µL of the secondary fluorescent QD-Abs (1:100 QD-Abs in 5% BSA), was captured by the drug-BSA present in the nitrocellulose membrane. Finally, in step (3), reservoir 3, which is filled with 10 µL of PBS, was used to wash the remaining fluorescence reagents in the detection zone. Note that a higher intensity than that in (**b**) is apparent in (**a**)

Figure 6 shows the microfluidic sequence for benzodiazepine detection. The same procedure is applied in both designs with and without the π-valves. The domino release idea from Yafia et al. ^28^ is appended to both circuits to increase the reliability of the release timing. The void area is connected by an air channel passing under the main channel to the other side. Except for the $\pi$-valve area, both designs (with and without the $\pi$-valve) are the same to guarantee a satisfactory comparison of the results. The end of the downstream channel is connected to an absorption pad with a suction pressure exceeding 5 kPa. Video 7 depicts the preprogrammed capillary-driven workflows used for benzodiazepine detection, with and without the $\pi$-valve. The immunoassay sequence is as follows: (0) introduction of 30 µL of the sample into the main channel with the anti-drug (0 or 1.0 µg/mL benzodiazepine and 5 µg/mL anti-benzodiazepine in 5% BSA) as the trigger solution; (1) reservoir 1 with 10 µL of 5% BSA for blocking; (2) reservoir 2 for the addition of 10 µL of the secondary QD-Abs (1:100 QD-Abs in 5% BSA); and (3) reservoir 3 with 10 µL of washing solution (PBS) to remove the remaining reagents in the detection zone. The BSA solution in the first reservoir washes the excess reagents coming from the solution of the main channel and, more importantly, provides a blocking buffer to avoid the nonspecific binding of the secondary antibodies in the next step. Steps 1 and 3 are positioned upstream of reservoir 2. This placement is crucial to avoid undesired binding of the secondary antibodies upstream of the circuit. These bindings can decrease their concentration and therefore their sensitivity while increasing the number of false-positives.

**Fig. 7:**
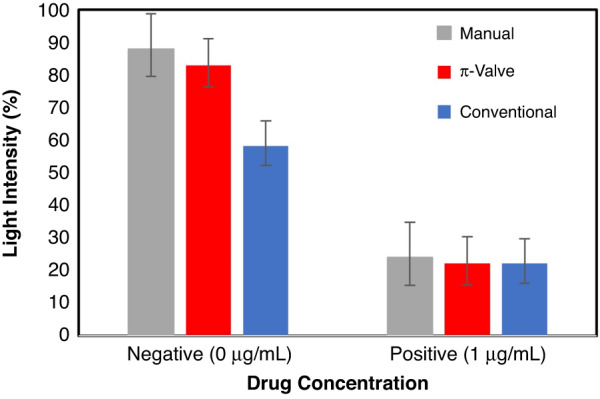
Relative fluorescence intensity versus drug presence. Red: the preprogrammed assay with a π-valve array. Blue: the preprogrammed assay without π-valves. Gray: outside microfluidics by manually pipetting into the nitrocellulose strip the same volume and chronological sequence. The nitrocellulose background light is the reference (0%). The standard deviation is determined from the measurement of three replicates

The results show that without a drug in the sample (a negative case), the fluorescence intensity is 40% higher with the π-valve than with the conventional valve (Fig. 7). This phenomenon occurs due to the loss of secondary antibodies (QD-Abs) within the circuit without π-valves by diffusion, leading to their dilution, adsorption to the adhesive, wasting as dead volumes, and their before due release earlier than trapping anti-drugs at the detection line/point. Video 8 shows possible diffusive phenomena during the microfluidic workflow based on conventional valves. SI 5 shows the presence of diffused QD-Abs in the circuit without π-valves, just before their preprogrammed release time. This phenomenon leads to less efficient immunoassays and even possible contamination of the subsequent wash, causing background noise. In the presence of 1.0 µg/mL benzodiazepine, light intensity decreases in all cases, indicating a positive test and the presence of a drug in the sample.

### Quantitative drug detection

**Fig. 8:**
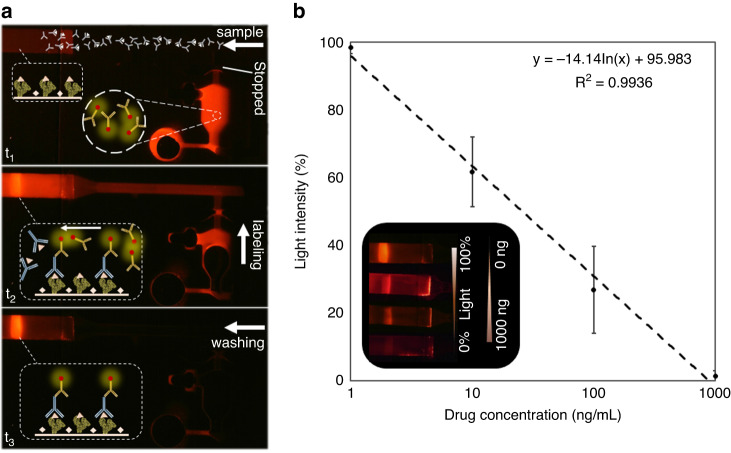
Quantitative competitive immunoassay for benzodiazepine using microfluidics with -valve. **a** Light evolution at the detection line by labelling and washing the blank sample. **b** Relative fluorescence intensity changes with the drug concentration within the sample. The maximum and minimum reference lights are considered for blank (0 ng/mL) to 1000 ng/mL (SD from three replicates) samples

Multiple quantitative drug detection processes (diazepam, which is a drug of the benzodiazepine family) are performed based on the same competitive immunoassay. However, instead of casting a drop of drug-BSA (benzodiazepine conjugated to BSA) on the nitrocellulose strip, it is linearly dispensed using an automated lateral flow reagent dispenser (ALFRD-Claremont) to achieve quantitative and reproducible results. Furthermore, the nitrocellulose is not sealed, readily displacing the air and liquid flows without forming a parabolic profile. SI 5 also depicts the difference arising from sealing the nitrocellulose. Figure 8 shows the change in fluorescence light intensity by drug concentration in samples from 1 to 1000 ng. The regression equation of y = −14.14 ln(x) + 95.98 represents the obtained data averages (3 replicates) per concentration. Video 9 displays fluorescence evolution at the detection line using the microfluidic device with *π*-valve for a blank sample.

## Discussion

Within the context of capillary-driven microfluidics and its point-of-care applications, multiple studies have paved the way for precisely manipulating reagents for approximately two decades^12^. We contributed to this effort by demonstrating the feasibility of utilizing an air gap between reagents as an efficient method for eliminating unwanted premixing and diffusion. However, as introducing air bubbles to fulfil this goal can lead to microfluidic malfunction^38^, the proposed valve was designed to displace and withdraw the air gap at a predefined time. Therefore, the valve isolated reagents and prevented cross-contamination without producing bubbles in circuits.

Due to the microfluidic chain reaction (MCR) of Yafia et al.^28^, the programmability of capillary flow was sufficient to encode complex immunoassay steps structurally. Moreover, very recently, suppressing undesired premixing between reagents by appending long U-turn liquid barrier channels to MCRs was reported^40^. Nevertheless, we aimed to avoid using long microchannels filled with liquids because, although they avoid reagent premixing, in our competitive immunoassay using fluorescence detection, diffusion of the QD secondary antibodies to these liquid channel barriers would reduce the immunoassay sensitivity. Thus, we moved into an adjustable air barrier ($\pi$-valve) as the desired solution to avoid unwanted diffusion. Covering the proposed valve both theory-wise and application-wise, we revealed a particular advantage to applying it to competitive immunoassays in which reagent diffusive premixing and inaccurate sequences could reduce the assay sensitivity.

To date, advanced additive manufacturing technology has made innovative solutions rapidly possible by applying 3D microfluidic features^41^. Utilizing this technology, a π-valve array was sequenced in a single autonomous device as a proof of concept to show the designed valve advantage for competitive drug detection. A considerable increase in the fluorescence intensity (40%) during the UV exposure process was detected for the circuit with a π-valve array compared to the same circuit without π-valve. Therefore, integrating π-valve as an appendix to the previously offered designs in the literature^12,28^ provided better control of the QD-Abs. Moreover, when using π-valves, we have not experienced a considerable increase in the response time (the entire detection process took less than half an hour). Therefore, the proposed valve showed aptitude as a tip-top supplement to level the sensitivity of capillary-driven-based assays; additionally, fast diagnostic tests could take advantage of this valve. In addition to the microfluidic system, we used a small LED-based portable microscope powered by a USB port for readout. As a result, the miniaturized detection system showed high potential for facile conversion into a POC device.

## Conclusions

A novel capillary valve that could avoid undesired cross-contamination before the valve activation time was designed and validated. The valve eliminated unwanted mixing and minimized sample waste after triggering. The diffusion of liquid molecules during valve function was studied and compared with a conventional valve. While the conventional valve experienced ~20% diffusion, it was completely eliminated in the π-valve. A sequence of 3 flow events using an array of 3 π-valves was demonstrated to be suitable for the sophisticated preprogramming of the reagent flows that were applicable in a point-of-care device for drug detection. By using the π-valve, the light intensity was enhanced by ~40% relative to the conventional valves. The π-valve could serve as a promising tool for conducting automated immunoassays in miniaturized detection systems because it reduces the cross-contamination between reagents and minimizes dead volumes.

## Supplementary information

Supplementary material

Video 1

Video 2

Video 3

Video 4

Video 5

Video 6

Video 7

Video 8

Video 9
