# Supplementary material for: Diffusion-free valve for preprogrammed immunoassay with capillary microfluidics
Source: Microsyst Nanoeng. 2023 Jul 17;9:91. doi: 10.1038/s41378-023-00568-2 (PMC10352302; doi:10.1038/s41378-023-00568-2)
Supplement: Supplementary file 1 — Supplementary material [file 41378_2023_568_MOESM1_ESM.docx]

DIFFUSION-FREE VALVE FOR PREPROGRAMMED IMMUNOASSAY WITH CAPILLARY MICROFLUIDICS

Pooya Azizian,^a,b^ Jasmina Casals-Terré,^b^ Jordi Ricart ^a^ and Joan M. Cabot ^a.^*

^a.^ Energy and Engineering Department, Leitat Technological Center, Terrassa (Barcelona) Spain.

^b.^ Mechanical Engineering Department, Technical University of Catalonia, Terrassa (Barcelona) Spain.

* Corresponding Author

Email: jmcabot@leitat.org; Tel: +34 937882300.

Supporting Information

**Videos:**

Video 1 – Numerical simulation of the $\pi$-valve function (MP4)

Video 2 – $\pi$-valve function in PDMS microfluidics (MP4)

Video 3 – $\pi$-valve function in 3DP microfluidics (MP4)

Video 4 – Modified $\pi$-valve for low resolution 3DP (MP4)

Video 5 – Valve effects on downstream and reagent loading (MP4)

Video 6 –The sequenced $\pi$-valve for preprogrammed microfluidics (MP4)

Video 7 – Automated microfluidics for benzodiazepine detection (MP4)

Video 8 – QD-Abs diffusion through a conventional valve (MP4)

Video 9 – Fluorescent light evolution at the detection line using $\pi$-valve. (MP4)

**Supporting text:**

SI 1 – Governing equations and numerical method (PDF)

SI 2 – Modified $\pi$-valve for low-resolution 3DP (PDF)

SI 3 – π-valve impacts on reagent loading and downstream sensing (PDF)

SI 4 – Preprogrammed liquid release by parallel π-valves (PDF)

SI 5 – Impacts on an immunoassay (PDF)

ACKNOWLEDGMENT

This project has received funding from the European Union’s Horizon 2020 research and innovation program under the Marie Skłodowska-Curie grant agreement no. 813863. This project has received funding from the European Union’s Horizon 2020 research and innovation program under the Marie Skłodowska-Curie grant agreement No 801342 (Tecniospring INDUSTRY).

All authors are joint inventors on a Patent Application PCT/ES2021/070720, which covers the π-valve concept (international publication number WO 2023/057659 A1).

1. **Governing equations and numerical method**

A set of governing equations considering incompressible multi-phase flow (immiscible - liquid-air interface) for capillary-driven microfluidics was expressed using: i) the conservation of momentum equation (Eq. 1); ii) the variable-density flow pattern (advection equation for density) (Eq. 2); iii) and the continuity equation (each fluid phase is considered to be incompressible) (Eq. 3).^1–4^The surface tension in Eq. 1 was simulated as a volumetric force acting on the fluid near an interface that is generated by the Continuum-Surface-Force method.^5^ $c(\boldsymbol{x},t)$ is defined as the volume fraction field for every cell of the numerical mesh to trace position of the interface. Then, the advection equation for the density can be replaced with an equivalent advection equation for the volume fraction (Eq. 4).^1,6^

| $\rho(\partial_{t}\mathbf{u}+\mathbf{u}.\nabla\mathbf{u})=-\nabla p+\nabla.(2\mu\mathbf{D})+\sigma\kappa\delta_{s}\mathbf{n}$ | (Eq. 1) |
| --- | --- |
| $\partial_{t}\rho+\nabla.(\rho\mathbf{u})=0$ | (Eq. 2) |
| $\nabla.\mathbf{u}=0$ | (Eq. 3) |
| $\partial_{t}c+\nabla.(c\mathbf{u})=0$ | (Eq. 4) |

where $\boldsymbol{u}=(u, v, w)$, $p$, and $\boldsymbol{D}$ are velocity vector, pressure, and deformation tensor defined as $\left( D_{ij}=\left( \partial_{i}u_{j}+\partial_{j}u_{i} \right)/2 \right)$, respectively.^1^ $\rho=\rho(\boldsymbol{x}, t)$ and $\mu=\mu(\boldsymbol{x},t)$ are density and viscosity of the fluid, respectively. $\delta_{s}$ is the Dirac delta function indicating that the surface tension force only acts at the interface, where its coefficient ($\sigma$) is focused. The interface curvature is specified as $\kappa$, and the unit vector perpendicular to the interface is specified as $\boldsymbol{n}$.^7^

The numerical method is based on the finite-volume discretization of the governing equations. The volume fraction function $c(\boldsymbol{x},t)$ for the volume of fluid (VOF) numerical method traces the multiphase interfaces. The relevant fluid properties, including viscosity ($\mu$) and density ($\rho$), are defined below where $\beta$ stands for the two-phase (Eq. 5) and three-phase (Eq. 6) flows.^6^

| $\beta\left( c_{1} \right)=c_{1}\beta_{1}+\left( 1-c_{1} \right)\beta_{2}$ | (Eq. 5) |
| --- | --- |
| $\beta\left( c_{1},c_{2} \right)=c_{1}\beta_{1}+c_{2}\beta_{3}+\left( 1-c_{1}-c_{2} \right)\beta_{2}$ | (Eq. 6) |

Indexes 1, 2, and 3 are related to three fluid phases. The surface tension force at the interface (Eq. 1) denotes the interaction of different fluid phases. Hence, the cells with multiphase interfaces are defined as a heterogeneous mixture. A piecewise-linear VOF scheme was employed to separate phases from each other. The interface in each cell is represented by a line (plane in 3D) defined as Eq. 7.^1,8^

| $\mathbf{m}.\mathbf{x}=\alpha$ | (Eq. 7) |
| --- | --- |

where $\boldsymbol{x}$ is position vector and $\boldsymbol{m}$ is the local normal vector to the interface. $\alpha$ is then determined assuming that the fluid volume fraction within each cell is equal to $c$.^1^

The governing equations were solved by Gerris open-source computational fluid dynamic (CFD) code using the adaptive mesh refinement (AMR) technique and Semi-Structure Quad/Octree spatial cells.^7,8^ AMR technique focuses on and makes the grids finer at the regions of importance (singularities, sharp corners, interfaces, etc.) where the gradients of the physical parameters are high.^3^ The AMR technique was examined with the maximum levels of refinement of 4, 5, and 6. A cell of level n has a resolution of $2^{n}$ in each coordinate. Also, 0 and n are the refinement levels of the root cell and the recursive descendant cells, respectively.^3,7^ The meniscus of a two-phase interface at the expanded junction varied approximately more than 10%, with one level increase in the superlative refinement from 4 to 5. But, increasing from 5 to 6 it varied less than 5%. Therefore, the grid refinement levels from 3 to 5 were applied in the numerical study of the valve, respectively, from the least to the highest phase gradient. Moreover, the height function method was used to incorporate the effect of contact angle as a representation of surface tension forces at contact lines within 3D-VOF.^9,10^

1. Popinet, S. An accurate adaptive solver for surface-tension-driven interfacial flows. *Journal of Computational Physics* **228**, 5838–5866 (2009).

2. Azarmanesh, M., Farhadi, M. & Azizian, P. Double emulsion formation through hierarchical flow-focusing microchannel. *Physics of Fluids* **28**, 032005 (2016).

3. Azarmanesh, M. *et al.* Passive microinjection within high-throughput microfluidics for controlled actuation of droplets and cells. *Sci Rep* **9**, 6723 (2019).

4. Azarmanesh, M., Farhadi, M. & Azizian, P. Simulation of the double emulsion formation through a hierarchical T-junction microchannel. *International Journal of Numerical Methods for Heat & Fluid Flow* **25**, 1705–1717 (2015).

5. Brackbill, J. U., Kothe, D. B. & Zemach, C. A continuum method for modeling surface tension. *Journal of Computational Physics* **100**, 335–354 (1992).

6. Azizian, P. *et al.* Electrohydrodynamic formation of single and double emulsions for low interfacial tension multiphase systems within microfluidics. *Chemical Engineering Science* **195**, 201–207 (2019).

7. Popinet, S. Gerris: a tree-based adaptive solver for the incompressible Euler equations in complex geometries. *Journal of Computational Physics* **190**, 572–600 (2003).

8. Chen, X. & Yang, V. Thickness-based adaptive mesh refinement methods for multi-phase flow simulations with thin regions. *Journal of Computational Physics* **269**, 22–39 (2014).

9. Afkhami, S., Zaleski, S. & Bussmann, M. A mesh-dependent model for applying dynamic contact angles to VOF simulations. *Journal of Computational Physics* **228**, 5370–5389 (2009).

10. Afkhami, S. & Bussmann, M. Height functions for applying contact angles to 3D VOF simulations. *International Journal for Numerical Methods in Fluids* **61**, 827–847 (2009).

1. **Modified π-valve for low-resolution 3DP**

The advent of 3D printing (3DP) has become a significant change in the field of microfluidics. The ability to fabricate a multilevel device in a single step has obvious advantages. However, the channel sizes and geometries may differ significantly from the CAD due to over-polymerization. Furthermore, the surface roughness is not homogenous between the channel walls (layer-by-layer roughness versus pixel-size polymerization). These defects change the capillary pressures and fluidic resistances and sometimes can cause device malfunction. This can be appreciated specially when 3D printers are used close to the limit of their resolution. It can also happen during the last prints after long printing periods (before the replacement of the resin bath). The main misfunctioning of the valve is that the liquid from the main channel did not go through the shallow branch completely. This was a problem because a small air gap inside the shallow channel can produce an air bubble and cause device malfunction. To increase tolerances between parts in the same batch and ensure device performance, a third branch was incorporated to avoid the formation of these bubbles. With the same dimensions as the shallow branch, this modification solved the problem while keeping the venting of the void chamber. **Figure S. 1** describes and illustrates the valve function. In this case, the red liquid was vented throw the second shallow branch so the first one can be filled completely and avoid the generation of bubbles. **Video 4** (SI) illustrates the activation and release of this π-valve.


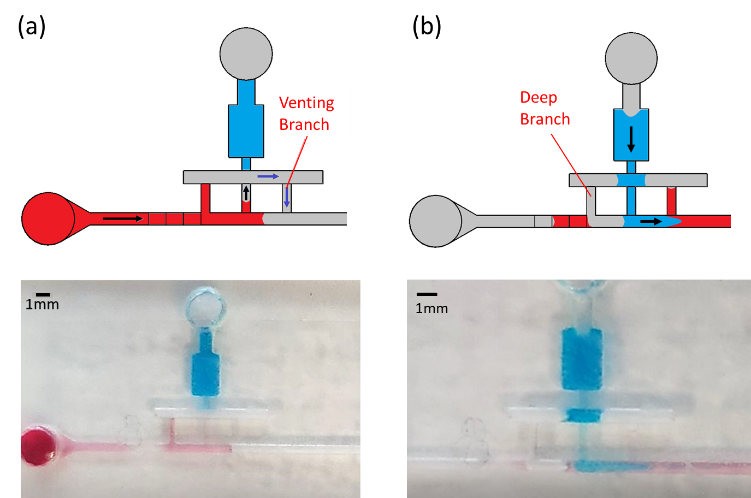


**Figure S. 1** The modified capillary 𝜋-valve. **(a)** The venting branch provides a path for the air to be displaced until the shallow branch is filled completely. **(b)** Pneumatic suction triggers the valve through the deep branch.

1. $\boldsymbol{\pi}$**-valve impacts on reagent loading and downstream sensing**

Precise reagent volume loading is important for reliable capillary-driven microvalves. The reagents are usually in μL range volumes, and even half a μL change can lead to a valve malfunction. For instance, **Figure S. 2. a** shows a reagent backflow from the main channel into the reservoir when the loading is incomplete. On the contrary, when the reservoir is slightly overloaded, there is considerable convection into the main channel before the programmed activation time (**Figure S. 2. b**). In the case of correct loading, where these conventions are diminished to diffusions, still, the valve activation mostly happens sooner than the desired programmed time. This is because inevitable surface defects generate uncertain resistances that can lead to a considerable dead volume in the main channel and even disrupt the flow sequence (**Figure S. 2. c**). However, using the proposed π-valve diminishes the precision required to load the reagent as well as a considerable decrease in the dead volume from μL to nL scale. **Video 5** demonstrates the consequences of inconsistent reagent loading, and how π-valve can avoid it (**Figure S. 2. a-c** corresponds to **Video 5. a-c**). The video shows an effective trigger, resulting in downstream sensing optimization. To check the robustness, we tested the π-valve when the volume was approximately twice more than the one required to fill the reservoir, also placing the chip vertically against gravity. Neither fully overloading nor gravity could induce valve failure (**Figure S. 2. d**). Furthermore, we successfully activated the π-valve using 2% Tween 20 (multiple times more than the surfactant concentration used for standard bioassays), whereas the conventional valves could handle 0.5%. This is due to the deeper channel of the void comparing the main channel also the trapped air. This combination provides an extra pressure barrier (approximately 300 Pa based on simulation) to hold the liquid till activation.

In this paper, we tested the diffusion analysis just downstream of the valve. The impacts on the flow pattern further downstream (for instance the sensing area) can vary from case to case. Generally, the medium and the geometry can cause flow pattern change, as well as manipulate fluid-diffusive phenomena such as Taylor dispersion. In our case, the detection medium is nitrocellulose, analogous to the lateral flow strip. As a porous medium, it can accelerate the initial wicking; however, its resistance significantly decreases the overall flow rate. Although it slows the assay, this velocity reduction favorably suppresses Taylor dispersion, approaching a lateral pattern. Moreover, this fall in flow rate can be done by raising viscosity, such as using higher concentrations of BSA, and an expansion in the detection site width. Also, a deeper valve junction restricts Taylor dispersion as it first pushes the released liquid slightly back into the main channel upstream. Here, for both the π-valve and the simple valve, the junction depth is more than the detection site and their upstream resistance. Employing these points, fluid dispersions within microchannels were limited and turned transient to about less than 1-8% of the valve function time (respectively for the lowest-highest flowrate). However, the dominant valve malfunctions from diffusive pre-mixing can lead to an undesired non-lateral flow pattern in the detection site, which can be avoided using a more elaborate valve. Tracing the color intensity on the nitrocellulose, in the absence of π-valve, illustrate a considerable deviation from the programmed order and a lateral flow pattern, proving the π-valve benefits not only for limiting mixing in the downstream lateral flow sensing but also for accurate sequencing (**Figure S. 2. e**).


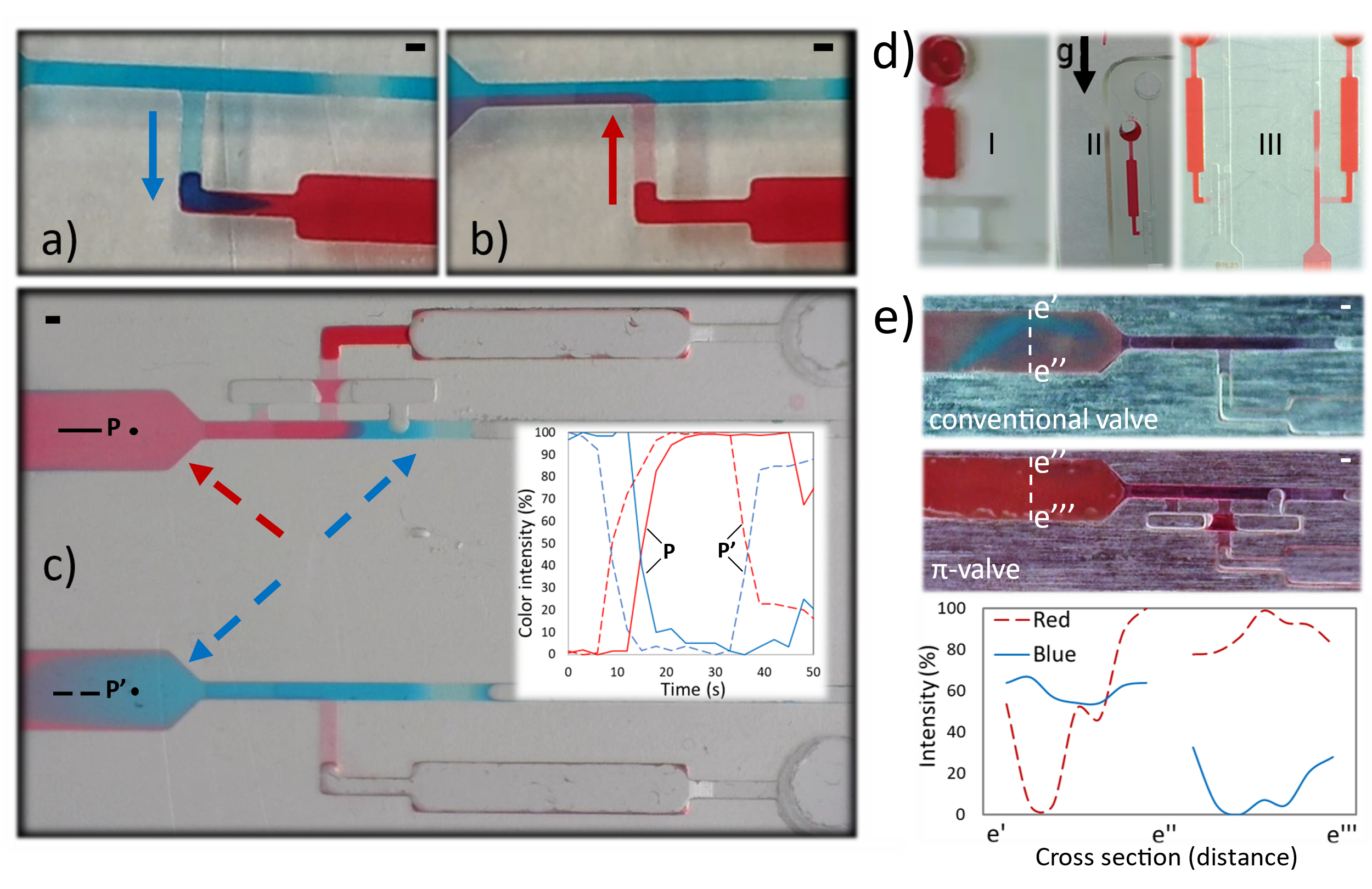


**Figure S. 2** Effect of downstream dispersion and loading on capillary-driven valves. **(a)** slightly lighter reagent loading leads to back-flow into the reservoir. **(b)** A slight overloading leads to convection into the main channel. (c) Red and blue color intensity versus time shows the π-valve benefit for better sequencing in the downstream sensing compared to the normal valve. **(d)** The valve certainty against overloading and surfactants (I: entrance overloading, II: against gravity, III: Effect of 0.5% Tween 20 on both valves). **(e)** Trace of the reagent colors on the nitrocellulose using microfluidics with and without π-valve. Scale bars are 500 μm.

1. **Preprogrammed liquid release by parallel** $\boldsymbol{\pi}$**-valves**

For the first sequencing strategy, the $\pi$-valves were connected in parallel to the main channel using different voids. **Figure S. 3** shows the release of 2 different fluids using 2 π-valves. The flow resistances were specifically designed to allow sequential activation times. Having different voids per valve is a limiting factor since they should be distributed along the main channel, and this increases the dead volume (red liquid in **Figure S. 3**). Furthermore, we would be limited in terms of capillary pressures and flow resistance. When we have multiple activation branches connected to the main channel, it increases the required activation suction pressure. It was noted that it could lead to corner flows, which diminish the valve function and take us away from the diffusion-free valve goal. So, for cases that require multiple π-valves, the second strategy using one deep branch was proposed in the manuscript.


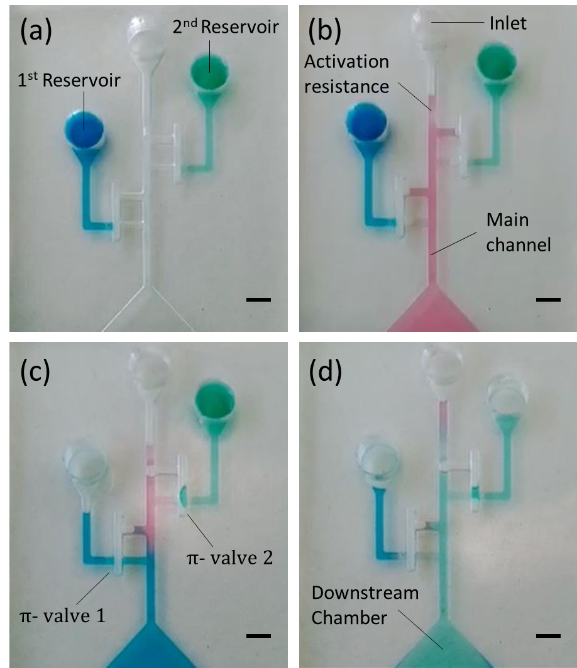


**Figure S. 3** Preprogrammed release of liquids using two π-valves with different channel resistances. **(a)** Fluid stopped at the two-level junctions. **(b)** Fluid was introduced into the main channel. Capillary force filled the channel with the red fluid, then emptied the liquid up to the resistance. **(c)** The π-valve with the less resistance was activated. When the blue reservoir was emptied, suction in the second void started. **(d)** The π-valve with higher resistance was activated, and the green liquid was released. Scale bar is 2 mm.

1. **Impacts on an immunoassay**

**Figure S. 4.** shows the fluorescent light intensity of the microfluidic devices used for the benzodiazepine detection immunoassay with and without π-valves, after the sample step and during the blocking, just before the second reservoir release (~5 mins after start). However, it can change for the microfluidic device without π-valve under various reagent loading conditions and downstream wicking. **Video 8** illustrates a possible reagent diffusive mixing during microfluidic sequencing using a conventional valve. Comparing **Figure S. 4. a** and **b** illustrates when there is no π-valve, the fluorescent secondary antibodies can diffuse into the circuit before the preprogrammed release time, and even slightly contaminates the subsequent washing step. **Figure S. 4. c** depicts a case where its washing step is highly contaminated without π-valve (I) compared to the same assay avoiding it having π-valve (II). But, this level of cross-contamination is not always the case depending on the manual reagent loading steps. This QD-Abs diffusion also can increase the possibility of adsorbing QD-Abs on the adhesive surface and leads to a decrease in the light intensity at the detection point/line where drug-BSAs are immobilized and concludes a false positive response of the competitive assay.

It should be mentioned that valve optimization is one of the impactful factors on an immunoassay, and optimizing other detection system components is as essential as them. For instance, an exploited method to advance a lateral flow pattern within nitrocellulose is, avoiding sealing there. It is due to a facile liquid flow through nitrocellulose and air displacement without forming a parabolic flow profile. **Figure S. 4. d** shows the difference between flow through the nitrocellulose when it is sealed (I) and it is not (II).


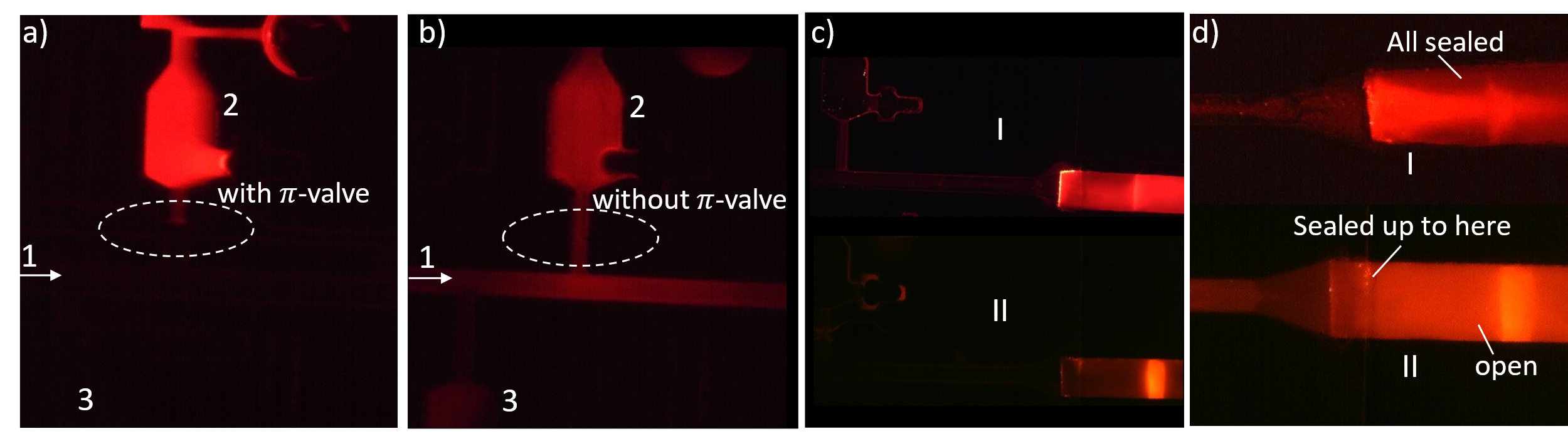


**Figure S. 4** Fluorescent light intensity shows the presence of the quantum dots conjugated antibodies (QD-Abs) within the microfluidic devices, including π-valves, and without π-valve during the first blocking step, just before the second reservoir release. **(a)** QD-Abs stayed inside the second reservoir because of the $\pi$-valve function. **(b)** QD-Abs diffused into the circuit upstream before their release time. Both the figures were taken using 1/8 sec camera exposure time and the equal fluorescent light power, which shows that because of the QDs-Abs diffusions, the light intensity of the microfluidics without the $\pi$-valve decreased. **(c)** When the washing step is contaminated without π-valve (I) compared to the same assay using π-valve (II). **(d)** Flow through the sealed (I) and not sealed (II) nitrocellulose shows the impacts of it on the flow pattern there.
